# Corynoxine isomers decrease levels of amyloid-β peptide and amyloid-β precursor protein by promoting autophagy and lysosome biogenesis

**DOI:** 10.1186/1750-1326-8-S1-P16

**Published:** 2013-09-13

**Authors:** Siva Sundara Kumar Durairajan, Yingyu Huang, Leilei Chen, Juxian Song, Liangfeng Liu, Min Li

**Affiliations:** 1 Neuroscience Laboratory, School of Chinese Medicine, Hong Kong Baptist University, Kowloon Jong, Hong Kong

## Background

One of the key histopathological features of Alzheimer’s disease (AD) is the formation of neuritic plaques characterized by accumulation of amyloid β-peptide (Aβ) derived from amyloid precursor protein (APP). Impairment of the autophagy-lysosomal degradation pathway has been associated with AD. Recently we have identified a novel natural autophagy inducer, corynoxine B (Cory-B), a major oxindole alkaloid from the Chinese medicinal plant *Uncaria rhynchophylla* (Miq.) Jacks (Gouteng in Chinese). In this study, we have also established that corynoxine (Cory) and its isomer of Cory-B, not only increases basal level of autophagy but also increases the lysosomal activity in neuronal cell lines. Interestingly Cory but not Cory-B showed the enhanced lysosomal activities.

## Materials and methods

N2a cell stably expressing Swedish APP (N2aSwedAPP) cells were treated with Cory or Cory-B at different dosage and time-course. Elisa was performed to measure the total Aβ in the supernatant of cell cutlture. Double immunostaining on N2aSwedAPP cells were performed using by carboxy terminal fragment (CTF) of APP (CT15) and LC-3 antibodies. Tg2567 mice at the age of 8 months, randomly distributed into Cory-B-treated Tg2567 group (Cory-B group) or Tg2567 group, and age matched non-Tg mice (C57BL/6, NT group) were assigned as aging control. Mice were intraperitoneally administered Cory-B (20 mg/kg/d) or vehicle (0.9% saline) once daily except weekends for 2 months. Cell and brain lysates were prepared for Western blot assay.

## Results

We observed a dose-dependent significant decrease in Aβ in cells incubated with Cory or Cory-B for 24h. Next we found that Cory- or Cory-B mediated decreases in Aβ are due to changes in its precursor protein APP, by measuring the level of full-length APP and APP-CTFs in cell lysates from N2a-sSwedAPP. Simultaneously, we also found that Cory and Cory B dose-dependently increased levels of LC3-II, an autophagy specific marker in N2aSwedAPP cells. Furthermore we found that Cory- or Cory-B induced LC3-II was enhanced by lysosome inhibitor chloroquine (CQ) and slightly decreased by autophagy inhibitor 3-methyladenine. We have confirmed this finding in immunocytochemistry of N2aSwedAPP cells with CT15 and LC3 antibody. Further, we found that only Cory but not Cory-B dose dependently increased the mature Cathepsin D. Cory treatment simultaneously increased Lamp-1 and decreased APP staining. Importantly, Cory dose-dependently induced the nuclear translocation of Transcription Factor EB (TFEB). In cell signaling studies, we found that Cory but not Cory-B time dependently decreased mTORC-1 activity via p70SK and GSK pathway. Two months of Cory-B treatment significantly decreased FI-APP and APP-CTF in Tg2567 (p<0.05). Cory-B treatment did not influence the levels of FI-APP in NT mice.

## Conclusions

Our data reveal that corynoxine isomers reduce Aβ through an increase of the degradation of APP and its CTF by activation of the autophagy/lysosomal pathway.

